# Evaluation of hippocampal *DLGAP2* overexpression on cognition, synaptic function, and dendritic spine structure in a translationally relevant AD mouse model

**DOI:** 10.1002/alz.70728

**Published:** 2025-09-30

**Authors:** Andrew Ouellette, Kristen O'Connell, Catherine Kaczorowski

**Affiliations:** ^1^ Graduate School of Biomedical Science and Engineering The University of Maine Orono Maine USA; ^2^ Genetics The Jackson Laboratory Bar Harbor Maine USA; ^3^ Department of Neurology The University of Michigan Ann Arbor Michigan USA

**Keywords:** aging, Alzheimer's disease, cognition, dendritic spine morphology, DLGAP2, electrophysiology, F1 hybrid 5XFAD, hippocampus, long‐term potentiation, postsynaptic density, synapse, synaptic plasticity

## Abstract

**INTRODUCTION:**

Developing effective therapeutics for Alzheimer's disease (AD) requires a better understanding of the molecular drivers of the disease. Our previous work nominated *DLGAP2* as a modifier of age‐related cognitive decline and risk for AD. We tested the hypothesis that overexpression of DLGAP2 in the hippocampus would protect against cognitive and synaptic deficits in a susceptible F1 5XFAD model.

**METHODS:**

*DLGAP2* was overexpressed in the hippocampus of F1 hybrid 5XFAD and non‐transgenic littermates using a viral approach. Cognitive function, electrophysiological properties, and dendritic spine morphology were assessed at 6 and 14 months of age.

**RESULTS:**

*DLGAP2* overexpression impaired synaptic plasticity and exacerbated AD‐related memory deficits but had minimal effect on spine structure or intrinsic neuronal properties.

**DISCUSSION:**

We highlight the complex role of *DLGAP2* in AD pathology. Targeted interventions involving postsynaptic proteins must consider potential adverse effects on synaptic integrity and cognitive performance, particularly in the context of AD.

**Highlights:**

- *DLGAP2* overexpression accelerates AD‐related impairment of contextual fear acquisition and memory.
- *DLGAP2* overexpression impairs synaptic plasticity prior to AD‐related memory impairment, but not intrinsic excitability.
- Effect of *DLGAP2* overexpression on thin spine density was blunted in AD mice from in vivo dendritic spine results that were replicated in cultured rodent neurons.

## 1 BACKGROUND

Alzheimer's disease (AD) is a progressive neurodegenerative disorder characterized by the accumulation of amyloid beta (Aβ) plaques, tau tangles, and significant neuronal/synapse loss, ultimately leading to cognitive decline. Although age is the most penetrant factor for the development of AD pathology, understanding the molecular underpinnings of the disease is critical for the development of targeted therapeutic strategies. Our previous cross‐species analysis identified the postsynaptic density (PSD) protein *DLGAP2* (Disks Large Associated Protein 2) as a potential mediator of AD‐related cognitive outcomes and showed that Religious Order Study and Memory and Aging Project (ROSMAP) AD patients with higher levels of DLGAP2 expression had improved late‐life cognitive outcomes.^1^ The *DLGAP2* protein is a key component in synaptic function and plasticity, organizing the PSD and mediating interactions between glutamate receptors and the actin cytoskeleton.^2^ Additionally, *DLGAP2* has been implicated in neurological disorders such as autism spectrum disorder and schizophrenia.^3^, ^4^, ^5^ However, its specific involvement in cognitive aging and AD remains poorly understood. Based on our previous work, we hypothesized that an increase in *DLGAP2* expression in memory‐relevant brain regions would minimize or prevent cognitive deficits associated with AD neuropathology.^1^ Therefore, in this study, we set out to test whether viral‐mediated overexpression of *DLGAP2* in the hippocampus would improve cognitive outcomes in the 5XFAD.D2F1 mouse strain with the highest polygenic risk score for human AD as well as being associated with lower cognitive resilience to AD in mice^6^, ^7^ and better understand the mechanisms in which *DLGAP2* may modify cognition and synaptic form/function.

## 2 METHODS

### 2.1 Animals

The B6‐5XFAD.D2F1 experimental mice and their non‐transgenic littermates (B6.D2F1) were produced by trio mating female 5XFAD mice on a congenic C57BL/6J background (034840, The Jackson Laboratory) with male DBA/2J (000671, The Jackson Laboratory). Mice were group housed and maintained on a 12‐h light/dark cycle with ad libitum access to food and water. All mouse experiments were conducted at The Jackson Laboratory in accordance with the National Institutes of Health Guide for the Care and Use of Laboratory Animals. All procedures and protocols were reviewed and approved by the Animal Care and Use Committee of The Jackson Laboratory (animal use summary number: 16049). A total of 161 female mice were used for this study. The number of mice needed per group was derived from a power analysis performed in GPower 3.1^8^; nine mice per group are needed to detect a 10% difference in spontaneous alternations on the Y‐maze task with ANOVA significance testing (alpha = 0.05, power = 0.85, three predictor variables, and eight groups). Based on our previous experience with surgical attrition and electrophysiology success rate, this number was doubled.

### 2.2 Viral constructs

Adeno‐associated viral (AAV) vectors were produced by Vector Biolabs (Philadelphia, PA, USA). *Dlgap2* cDNA and enhanced green fluorescent protein (eGFP) controls were both packaged into AAV serotype 9 vectors and stored in a 1X PBS buffer containing 5% glycerol at a 1.1 × 10^13^ GC/mL titer. Protein expression in both vectors was driven by a CamKII promoter and had a hemagglutinin (HA) tag fused to the C‐terminus of the expressed protein. The resulting vectors were subjected to purification by two rounds of CsCl density gradient centrifugation combined with ultracentrifugation to separate empty and full capsids.

### 2.3 Intrahippocampal injections

At 4 months of age mice were anesthetized under 4% to 5% inhalable isoflurane gas and positioned in a stereotaxic frame, then maintained at 1% to 2% inhalable isoflurane gas. Two holes were drilled into the skull at the following coordinates: −1.9AP, ± 1.4 ML, −1.6 DV. A microinjector attached to a 33‐gauge Hamilton syringe and syringe pump were used to deliver 500 nL of virus per hemisphere at a rate of 200 nL/min. Microinjectors were left in place for 5 min after injection to allow sufficient viral diffusion. Following injection, the cut was sutured using absorbable sutures. After surgery, topical bupivacaine was applied, and mice were monitored daily for at least 3 days. Viral spread was limited to the hippocampus/entorhinal cortex and did not spread to the cortex (Figure S1).

### 2.4 Y‐maze working memory task

All mice underwent the Y‐maze spatial working memory task at 6 months of age, and the aged cohort underwent a second trial at 14 months. Mice were allowed to freely explore a Y‐maze apparatus with three equally sized arms for 8 min: arm length of 30 cm, arm width of 5 to 6 cm, wall height of 12 to 18 cm. Working memory was measured by the percentage of spontaneous alternations (Number of spontaneous alternations/Total arm entries) between Y‐maze arms. Recorded videos were analyzed using the ANY‐maze behavioral tracking software (Stoelting Co., IL, United States). To reduce false‐positive arm entries, we defined an entry as occurring when 99% of the mouse's body (excluding the tail) entered a new arm. We excluded data from six animals that did not make at least six arm entries, the minimum required for four spontaneous alternations, from percentage spontaneous alternation data analysis. Distance traveled during testing (in meters) was also recorded as a metric of general locomotor activity.

### 2.5 Contextual fear conditioning

At 6 months (mo) of age, all mice underwent contextual fear conditioning (CFC) to assess the acquisition and recall of hippocampal‐dependent memory.^9^ On the first day of training, mice were placed in a training chamber, and four foot shocks (0.9 mA, 1 s) were delivered after a 150‐s baseline period. Four post‐shock intervals were defined as the 40 s following the end of each foot shock, and the percentage of time spent freezing during each interval was determined using FreezeFrame software (Actimetrics Inc., IL, USA). The percentage of time spent freezing following the final shock was used as a measure of contextual fear acquisition across the panel. Twenty‐four hours after training, mice were placed back into the training chamber, and the percentage of time spent freezing throughout the entire 10‐min test was measured as an index of contextual fear memory recall; no shocks were delivered during the testing session. In total, 78 mice were sacrificed after testing at 6 months of age; 72 mice were aged until 14 months. At 14 months of age, the aged cohort was placed into the conditioning chambers for 10 min with no foot shocks to determine that memory of their initial training regimen had been extinguished. One week later these mice underwent the same training and test protocol as their initial 6‐month timepoint. Outcomes from these tests were used as indicators of aged memory acquisition and recall.

### 2.6 Electrophysiology recordings

Mice were anesthetized with isoflurane before being sacrificed for electrophysiology recordings at 0 to 3 days after terminal CFC testing LTP outcomes were tested to ensure they were not confounded by the delay between CFC and harvest (F[3, 89] = 1.17, *p* > 0.05). Brains were rapidly removed and placed in ice‐cold cutting artificial cerebrospinal fluid (aCSF) containing (in mM) 93 NMDG, 2.5 KCl, 1.4 NaH2PO4, 30 NaHCO3, 20 HEPES, 25 D‐Glucose, 3 myoinositol, 5 sodium l‐ascorbate, 2 thiourea, 6.1 NAC, 3 sodium pyruvate, 0.01 taurine, 10 MgSO4.7H2O, and 0.5 CaCl2.2H2O; pH was adjusted to 7.4 and osmolarity to 300 mOsm. Acute transverse hippocampal slices (300 µm) were cut with a vibratome (Leica, VT1000S) in ice‐cold cutting aCSF. Slices were incubated at 34°C in carbogen bubbled cutting aCSF for 18 min. Slices were held at room temperature for 1 to 4 h before recording in holding aCSF containing (in mM) 94 NaCl, 2.5 KCl, 1.4 NaH2PO4, 25 NaHCO3, 20 HEPES, 25 d‐Glucose, 3 myoinositol, 2 thiourea, 5 sodium l‐ascorbate, 6.1 NAC, 3 sodium pyruvate, 0.01 taurine, 2 MgSO4.7H2O, and 2 CaCl2.2H2O; pH was adjusted to 7.4 and osmolarity to 300 mOsm. Whole‐cell current clamp recordings were made in CA1 pyramidal neurons at 34°C under visual guidance of a video microscope (Olympus, Moment CMOS camera) using patch pipettes with a resistance of 3 to 5 MΩ filled with potassium gluconate‐based internal solution (in mM): 126 K‐Gluconate, 4 KCl, 10 HEPES, 4 MgATP, and 0.3 NaGTP, 10 Na‐phosphocreatine, 0.2% biocytin; pH was adjusted to 7.4 and osmolarity to 290 mOsm. Recordings were performed in an aCSF solution containing (in mM), at 34°C, 119 NaCl, 2.5 KCl, 2.5 CaCl2.2H2O, 1 MgSO4, 1.25 NaH2PO4, 23 NaHCO3, and 10 d‐glucose. A concentric bipolar stimulating electrode (FHC, Bowdoin, ME, USA) connected to an A365 stimulus isolator (WPI, Sarasota, FL, USA) was placed in the tissue along the Schaffer collateral 50 to 200 µm away from the patched cell's soma. A Multiclamp 700B amplifier, pClamp 11.2.1 software, and Digidata 1550B interface (Molecular Devices) were used to acquire data. Recordings were acquired at a 10‐kHz sampling frequency and digitized at 20 kHz in current clamp mode. Neuron membrane potential was held at −67 mV. Series resistance and capacitance were monitored and compensated throughout the recordings. Neurons with >40 MΩ series resistance were excluded. Changes in input resistance were measured by injecting 1s square step current into the soma ranging from −50 to 50 pA in 10‐pA intervals, and input resistance was calculated in Easy Electrophysiology. Action potential threshold current was measured by injecting a 2‐ms current step into the soma ranging from 200 to 600 pA; the minimum current needed to elicit an action potential was recorded as the threshold current. After‐hyperpolarization (AHP) was initiated by a 25‐action potential burst. Medium afterhyperpolarization (mAHP) was measured as the peak negative membrane potential relative to baseline, and the slow afterhyperpolarization (sAHP) was measured as the average negative membrane potential relative to baseline at 1 to 1.05 s after the last brief current injection of protocol for triggering post‐burst AHP.^10^

**RESEARCH IN CONTEXT**

1. **Systematic review**: The authors reviewed the literature using PubMed and recent publications to investigate DLGAP2, a PSD protein implicated in synaptic function, dendritic spines, and social disorders such as autism spectrum disorder and schizophrenia. Relevant findings regarding synaptic protein modulation and cognition are cited.
2. **Interpretation**: This study shows that hippocampal DLGAP2 overexpression impairs synaptic plasticity and subsequently worsens contextual fear memory deficits in 5XFAD mice. These findings suggest that while DLGAP2 may be critical for normal synaptic function, its overexpression can negatively impact cognition in the presence of AD mutations that drive amyloid neuropathology.
3. **Future directions**: Future research should explore the mechanisms by which DLGAP2 overexpression disrupts synaptic and cognitive functions, with key areas including (a) interactions between DLGAP2 and other postsynaptic proteins, (b) age‐ and pathology‐dependent effects of DLGAP2, and (c) the therapeutic potential of modulating DLGAP2 expression to improve cognitive outcomes in AD models. Knockdown of DLGAP2 will be tested for efficacy in F1 hybrid 5XFAD animals.

Synaptic throughput was assessed with input/output curves by applying a standardized current ramp to the Shaffer collateral starting at 25 µA, proceeding to 50 µA, then increasing up to 600 at 50‐µA intervals every 20 s. Excitatory postsynaptic potentials (EPSPs, mV) were recorded in response to each stimulus. The current needed to elicit a 3‐ to 5‐mV response was used for paired‐pulse ratio (PPR) and LTP recordings. EPSPs were monitored during a 5‐min baseline period, with one EPSP evoked every 20 s. To induce synaptic plasticity, a theta‐burst stimulation (TBS) protocol was used that consisted of theta‐patterned synaptic activation (five stimuli at 100 Hz) of proximal neuronal inputs repeated at 5 Hz for 3 s. EPSPs were then evoked for 35 min every 20 s; the peak amplitude of each EPSP relative to baseline membrane potential was recorded. These peak amplitudes were aggregated into 5‐min bins for analysis. To calculate the PPR, two stimulus pulses were delivered at 25‐, 50‐, 75‐, or 100‐ms intervals, and the PPR was measured by dividing each EPSP by the first.

### 2.7 Immunohistochemistry (IHC)

Immediately after each electrophysiology recording was complete, brain slices were placed in a 4% paraformaldehyde (PFA) solution for 24 h and then transferred to 1xPBS containing 0.1% sodium azide. Slices were incubated with rabbit anti‐HA antibody (Cell Signaling, Catalog No.: C29F4) and streptavidin‐Alexa 633 (Thermo Fisher Scientific, Catalog No.: S21375) at 1:1000 dilution each for 24 h at 4°C. After washing, slices were incubated in 1:500 goat anti‐rabbit AlexaFluor 568 (Thermo Fisher Scientific, Catalog No.: A‐11036) for 2 h at room temperature. During the final wash prior to mounting, slices were incubated with 1:2000 DAPI for 10 min. Slices were mounted on Colorfrost Plus slides (Fisherbrand) with 0.36‐mm spacers and SlowFade Glass Antifade Mountant (Invitrogen). Slides were stored in the dark at 4°C.

### 2.8 Image acquisition

Confocal microscopy was used to capture images of biocytin‐filled dendrites from patched CA1 pyramidal neurons. Imaging was performed by a blinded experimenter on a Stellaris 5 confocal microscope (Leica. Wetzlar, Germany) using a Plan Apo 63×/1.40NA oil‐immersion objective. Three‐dimensional z‐stacks were obtained of secondary dendrites from filled neurons that met the following criteria: (1) within 80 µm working distance of microscope, (2) relatively parallel to the surface of the transverse section, (3) no overlap with other branches, and (4) located between 50 and 150 µm from the soma. Up to five apical and basal dendrites were acquired per cell. Dendrite segment images were acquired in z‐stacks with a step size of 0.13 µm, image size of 1024 × 512px, zoom of 4.8×, line averaging of 4×, and acquisition speed of 400 Hz; individual pixel size was 0.36 × 0.36 um.

### 2.9 Dendritic spine reconstruction

Confocal z‐stacks of dendrites were deconvolved using Huygens Deconvolution System (16.05, Scientific Volume Imaging, the Netherlands) using the following settings: deconvolution algorithm: GMLE; maximum iterations: 10; signal‐to‐noise ratio: 15; quality: 0.003. Deconvolved images were saved in.tif format. Deconvolved image stacks were imported into Neurolucida 360 (2.70.1, MBF Biosciences, Williston, VT, USA) for dendritic spine analysis. For each image, the semi‐automatic voxel scoop algorithm was used to trace the dendrite. All assigned points were examined to ensure they matched the dendrite diameter and position in the *X*, *Y*, and *Z* planes and were adjusted if necessary. Dendritic spine reconstruction was performed automatically using a voxel‐clustering algorithm, with the following parameters: outer range = 5 µm, minimum height = 0.3 µm, detector sensitivity = 80%, and minimum count = 8 voxels. The automatically identified spines were examined to ensure that all identified spines were real and that all existing spines had been identified. If necessary, spines were added by increasing the detector sensitivity to 100% and manually identified. Merge and slice tools were used to correct errors made in the morphology and backbone points of each spine. Each dendritic spine was automatically classified as a thin spine, stubby spine, mushroom spine, or filopodia spine based on constant parameters. Three‐dimensional dendrite reconstructions were exported to Neurolucida Explorer (2.70.1, MBF Biosciences, Williston, VT, USA) for branched structure analysis, which provides measurements of dendrite length; number of spines; spine length; number of thin, stubby, and mushroom, and filopodia spines; spine head diameter; and spine neck diameter, among other measurements. Spine density was calculated as the number of spines per 10 µm of dendrite length. Values for each mouse were calculated by averaging all corresponding dendrites. Statistical analyses were performed using mice as the independent unit. Values for apical and basal dendrites were averaged separately per mouse. Number of animals, cells and dendrites imaged are listed in Table 1.

**TABLE 1:** Sample *n* sizes are listed for the number of animals allocated to each group at the start of study, behavior, electrophysiology, and dendritic spine measurements. CFC was longitudinally measured, so the 14‐month cohort is included in the 6‐month data. For electrophysiology measurements, the *n* size is reported as mouse *n*/cells *n*. For dendritic spine data, *n* sizes are reported as mouse *n*/cells *n*/dendrite *n*.

Age (months)	Genotype	AAV status	Allocated mice	CFC	Y‐maze	Input/ output	LTP	PPR	AP current threshold	Input resistance	AHP	Apical dendrites	Basal dendrites
6	Ntg	Control	20	43	37	7/13	6/10	7/11	7/11	7/13	7/13	6/8/20	4/4/6
6	Ntg	Dlgap2	19	41	36	6/12	4/7	6/10	6/13	6/12	6/12	6/6/12	3/3/8
6	5XFAD	Control	20	35	32	7/12	5/7	7/12	7/12	7/12	7/12	5/6/25	5/6/15
6	5XFAD	Dlgap2	19	35	33	8/12	6/9	8/12	8/12	8/12	8/12	8/10/32	6/8/18
14	Ntg	Control	24	24	24	8/14	7/11	9/14	9/15	9/15	9/15	7/9/31	7/9/19
14	Ntg	Dlgap2	26	24	24	11/20	11/19	11/20	11/20	11/20	11/20	10/15/57	10/12/31
14	5XFAD	Control	16	13	13	9/16	9/16	8/13	9/16	9/16	9/16	6/9/30	6/8/14
14	5XFAD	Dlgap2	17	11	11	8/13	8/11	7/10	8/14	8/13	8/13	7/9/30	4/5/11

Abbreviations: AAV, adeno‐associated virus; AP, action potential; APH, after‐hyperpolarization; CFC, contextual fear conditioning; LTP, long‐term potentiation; PPR, paired‐pulse ratio.

## 3 RESULTS

### 3.1 Experimental design and AAV validation

**FIGURE 1:**
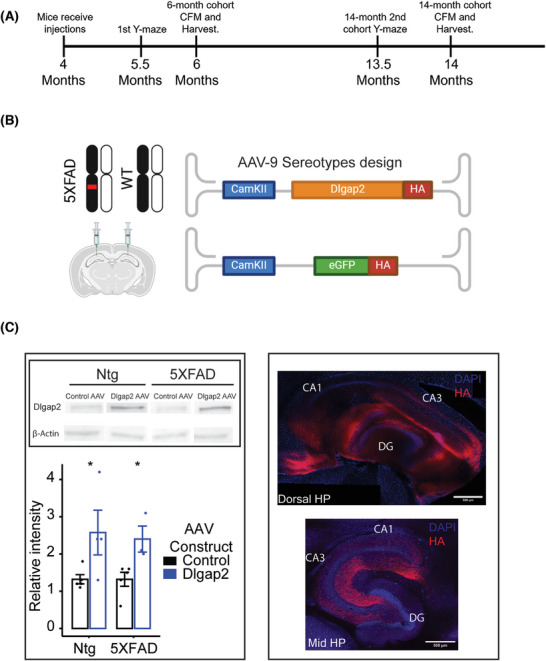
AAV design and study overview. (A) Timeline of experiments showing age of mice in month starting at time of injection until terminal 6‐ and 14‐month timepoints. (B) 5XFAD and WT animals were injected with an adeno‐associated viral (AAV)‐9 construct with either DLGAP2 or enhanced green fluorescent protein (eGFP) cDNA behind a CamKII promoter into the CA1 hippocampus. (C) Left: Western blot results showing levels of DLGAP2 protein expression in whole hippocampal sections from 14‐month‐old mice injected with either eGFP controls or Dlgap2 cDNA. The intensity of DLGAP2 bands were normalized to β‐Actin loading controls, *n* = 3 to 5 per group. Right: spatial visualization of exogenous DLGAP2 in 14‐month‐old animals. In general, viral spread was mostly observed in dorsal CA regions, dentate gyrus, and subiculum, with the highest expression seen in tracts projecting into more ventral CA1. *p < 0.05. CA, Cornu Ammonis.

An AAV‐9 containing *Dlgap2* cDNA fused to an HA epitope behind a CamKII promoter was used to drive overexpression of DLGAP2 in neurons of the hippocampus (Figure 1A–B). To verify that AAV successfully overexpressed *DLGAP2* in mice, we quantified *DLGAP2* protein expression via Western blot of injected hippocampal samples. *DLGAP2* expression was significantly elevated in animals injected with *Dlgap2* AAV compared to eGFP controls at 14 months of age in both non‐transgenic (Ntg) (mean change = 1.3, *t*[7] = −2.30, *p* < 0.05) and 5XFAD animals (mean change = 1.1, *t*[6] = −3.01, *p* < 0.05) (Figure 1C, Left, Figure S2), indicating that *DLGAP2* is robustly overexpressed within the hippocampus. IHC in a subset of mice confirmed that AAV expression was largely in dorsal CA1 regions, dentate gyrus (DG), and subiculum, and more ventral CA3. Expression was also observed along CA3–CA1 tracts in the medial hippocampus (Figure 1C right), but not in pyramidal cells originating from this layer.

### 3.2 DLGAP2 overexpression has no effect on spatial working memory

At 6 months of age, all mice were tested for spatial working memory using the Y‐maze task; the aged cohort was retested at 14 months. All groups performed above chance (50% SA), indicating non‐random exploratory behavior. Although we detected a statistically significant main effect of AAV construct on percentage of spontaneous alternations (F[1, 27] = 5.98, *p* < 0.05), there were no significant post hoc comparisons after correcting for multiple comparisons (Figure S3). These results, along with limited effect sizes, suggest that hippocampal *DLGAP2* overexpression does not significantly impact spatial working memory, regardless of age or AD status.

### 3.3 DLGAP2 overexpression is detrimental to AD‐related contextual fear memory decline

**FIGURE 2:**
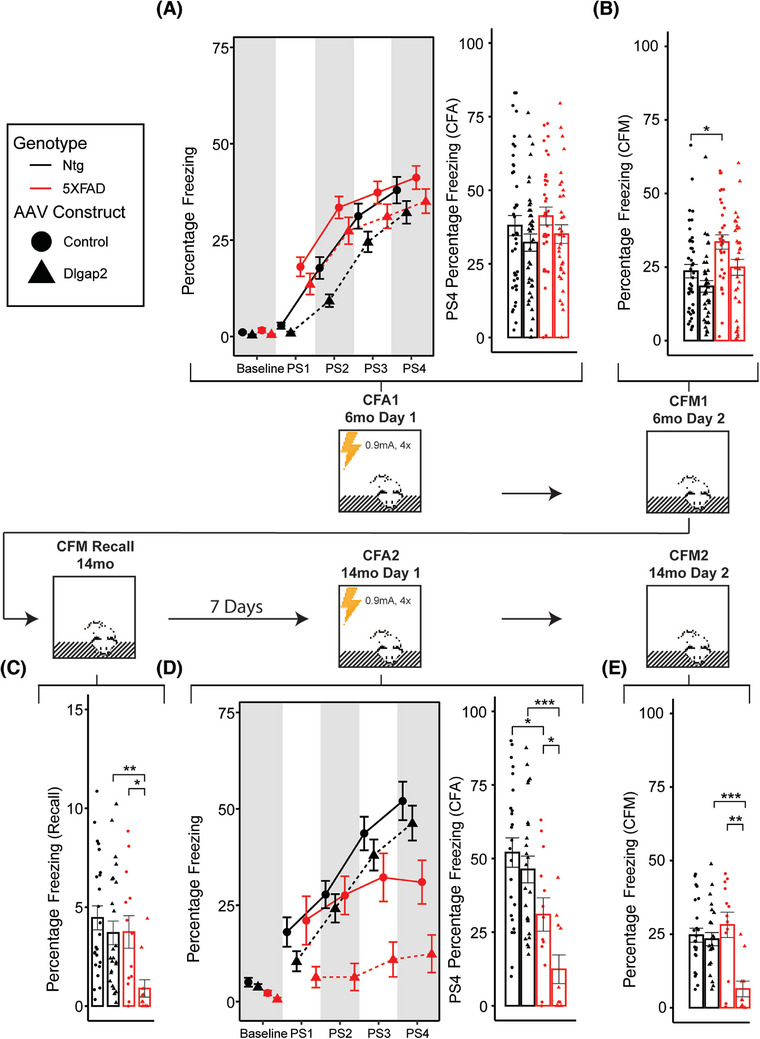
DLGAP2 overexpression impairs memory outcomes in aged 5XFAD animals. (A) Left: memory acquisition curves measured by percentage of time spent freezing during the 150‐s baseline period before shocks were administered and each 40‐s post‐shock interval. Right: 6‐month contextual fear acquisition measured (CFA) as the percentage of time spent freezing during 40‐s post‐shock 4 (PS4) interval. (B) 6‐month contextual fear memory (CFM) recall measured by percentage of time freezing during testing session with no shock administration 24 h after training. (C) Seven days before 14‐month retrain/testing, animals were placed in the chamber with no shocks and total percentage of freezing was measured as an indicator of how well they remembered the initial 6‐month training session. 5XFAD animals have a slight reduction in the percentage of freezing compared to controls. (D) Left: memory acquisition curves during retraining of 14‐month‐old animals. Right: CFA outcomes in 14‐month‐old animals during retraining session; 5XFAD animals overexpressing DLGAP2 have severe impairment in their ability to learn the task compared to controls. (E) CFM outcomes in 14‐month‐old animals 24 h after retraining. CFM was significantly impaired by DLGAP2 overexpression in 14‐month‐old 5XFAD animals. Error bars are represented as standard error around the mean. *P* values were derived from a *t*‐test and corrected for multiple testing error using the Bonferroni method (*p < 0.05, **p < 0.01, ***p < 0.001). *sample sizes* are listed in Table 1.

To determine whether DLGAP2 overexpression could ameliorate AD‐related cognitive deficits, we employed a novel longitudinal CFC paradigm at both 6 and 14 months of age. Age‐dependent memory acquisition and recall were assessed in all animals by measuring contextual fear acquisition (CFA) and contextual fear memory (CFM), respectively. We first established that initial training and testing in the 6‐ and 14‐month cohorts were comparable at their initial 6‐month timepoint; three‐way ANOVA indicates that both cohorts have comparable training/testing outcomes at 6 months (Figure S4). Two‐way repeated‐measures ANOVA detected a significant main effect of AD status on CFA where animals with the 5XFAD transgene tended to perform worse than Ntg counterparts, especially by 14 months of age (F[1, 63] = 9.68, *p* < 0.01). We detected a significant interaction between AD status × AAV construct (F[1, 63] = 19.84, *p* < 0.001) where AD animals overexpressing *DLGAP2* had worse outcomes compared to control injections. We observed a similar pattern in CFM outcomes; two‐way repeated‐measures ANOVA detected a significant decrease in *DLGAP2*–overexpressing mice compared to control injections (F[1, 57] = 6.63, *p* < 0.05) and a decrease from 6‐ to 14‐month age points (F[1, 57] = 10.12, *p* < 0.01), alongside significant interactions between AD status × age (F[1, 57] = 23.78, *p* < 0.001) (Figure S5), and AAV construct × age (F[1, 57] = 4.84, *p* < 0.05). At 6 months there was no significant effect of *DLGAP2*overexpression on CFA or CFM outcomes (Figure 2A,B). Interestingly, we observed an increase in CFM in 5XFAD control AAV animals compared to Ntg controls that was not observed in *DLGAP2* overexpressing animals.

To track individual changes in cognitive longevity, CFC was performed longitudinally in the 14‐month cohort. Therefore, we tested whether any experimental mice had retained memory of the initial 6‐month testing phase and whether this possible retention could affect retesting outcomes by measuring percentage freezing without foot shocks 1 week before terminal retraining/testing. Overall, animals had reduced memory recall compared to their 6‐month testing session, which indicates that by 14 months of age animals do not retain the memory of their initial training. Additionally, we found that 5XFAD animals overexpressing *DLGAP2* had significantly lower memory recall than Ntg and control AAV counterparts, suggesting that *DLGAP2* overexpression may have impaired long‐term memory recall (Figure 2C) (T[1,18] = 3.05, *p* adjusted < 0.05). However, the small effect size limits the interpretations that observed differences in CFA and CFM are confounded by prior memory retention.

By 14 months of age, 5XFAD animals displayed an expected decrease in CFA compared to Ntg controls (T(1,21.9) = 2.48, *p* adjusted < 0.05) (Figure 2D).^11^ However, we observed that *DLGAP2* overexpression was detrimental to CFA outcomes in 5XFAD animals. Slope acquisition curves showed that aged 5XFAD animals overexpressing *DLGAP2* were unable to learn the context of their environment and that their acquisition of the post‐shock 4 interval was severely impaired (Figure 2D). These data suggest that previously observed AD‐related memory acquisition deficits are exacerbated with *DLGAP2* hippocampal overexpression. Additionally, overexpression of *DLGAP2* reduced CFM performance in 14‐month 5XFAD animals compared to Ntg controls (Figure 2E). In contrast to CFA, we did not observe a significant decrease in CFM in 14‐month 5XFAD control AAV animals compared to Ntg controls, suggesting that *DLGAP2* overexpression accelerates AD‐related impairment of CFM at 14 months of age. Since CFC outcomes can be confounded by changes in baseline locomotor activity, similar to those we observed in the Y‐maze,^12^, ^13^ we analyzed 150 s of baseline freezing before shocks were administered on the training session across each experimental group. Two‐way repeated‐measures ANOVA detected significant main effects of AD status (F[1, 63] = 8.36, *p* < 0.01), age (F[1, 63] = 29.3, *p* < 0.001), and an interaction between age × AD status (F[1, 63] = 14.85, *p* < 0.001); no significant main effect of *DLGAP2* overexpression was detected. Additionally, post hoc analysis revealed that baseline freezing was reduced only in 14‐month 5XFAD animals that had been injected with *DLGAP2* AAV compared to Ntg counterparts (Figure S6B–F). Age also had a robust effect on baseline freezing, where Ntg 6‐month animals had significantly lower percentage freezing than 14‐month animals in both control and *DLGAP2*‐overexpressing cohorts. This age‐related change was not present in 5XFAD animals, suggesting that AD pathology blunts the natural tendency for increased locomotor activity as animals mature. These results suggest that AD‐related pathology may elevate locomotor activity and was exacerbated by *DLGAP2* overexpression but does not necessarily coincide with observed cognitive deficits.

### 3.4 DLGAP2 overexpression affects synaptic properties in presymptomatic 5XFAD animals

**FIGURE 3:**
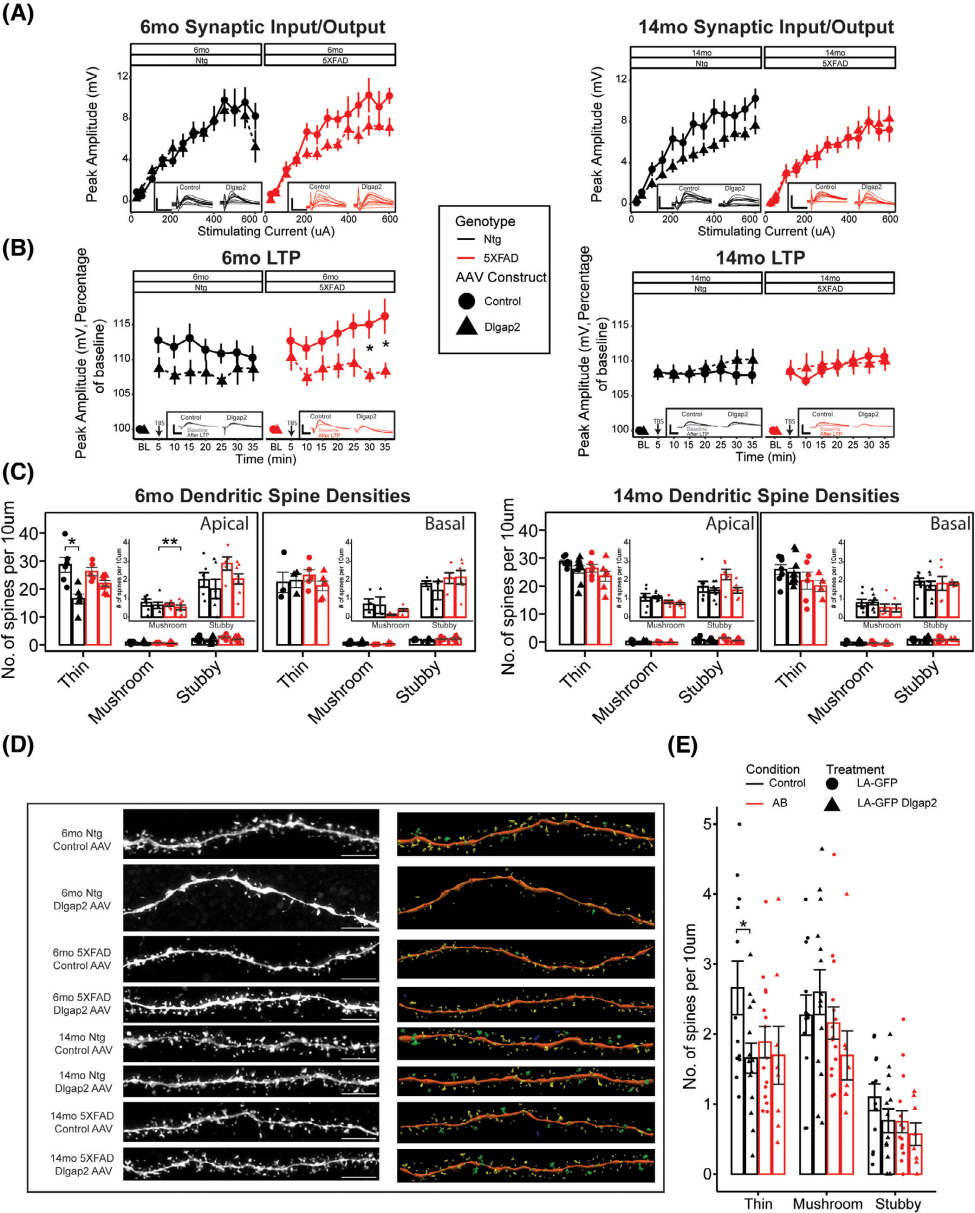
Synaptic functional and structural properties exhibit DLGAP2 overexpression, amyloid burden, and age interactions. (A) Input/output curves, stimulating current injected into Schaffer collateral are plotted against evoked excitatory postsynaptic potential (EPSP) amplitude of patched CA1 pyramidal cells for 6‐month‐old (right) and 14‐month‐old (left) animals. Insets show representative traces (scale bar: 10 ms, 15 mV), no main effect of DLGAP2 overexpression was observed. (B) Long‐term potentiation (LTP) of patched CA1 pyramidal cells, peak amplitudes of evoked EPSPs, normalized to baseline recordings, are plotted along the 35‐min time course in 5‐min bins for 6‐month‐old (right) and 14‐month‐old (left) animals. Inset with representative traces (scale bar: 10 ms, 20 mV), 6‐month‐old 5XFAD DLGAP2‐overexpressing animals had diminished LTP compared to controls. (C) Barplots showing dendritic spine density as number of thin, mushroom, or stubby spines per 10 µm of dendrite. Each dot represents a single cell; values from dendrites of that cell are averaged together with apical and basal dendrites kept separate. Plots are inset with rescaled *y*‐axis to show effect sizes within mushroom and stubby spines. Apical thin spines are reduced in Ntg 6‐month‐old DLGAP2‐overexpressing mice compared to mice injected with control adeno‐associated virus. (D) Representative images and reconstructions from captured apical dendrites for each experimental group. Spine types are visualized by color (yellow = thin, green = mushroom, red = stubby, and blue = filopodia). Scale bar: 5 µm. (E) Barplots showing dendritic spine density as number of thin, mushroom, or stubby spines per 10 µm of dendrite from cultured rat hippocampal neurons at 14 days in vitro. Each dot represents a single cell. Thin spines are reduced in cultures overexpressing Dlgap2 with no amyloid beta (*n* = 8 to 15 cells per group). *P* values were derived from a *t*‐test and corrected for multiple testing error using the Bonferroni method (*p < 0.05, **p < 0.01, ***p < 0.001). Error bars are represented as standard error around the mean. S*ample sizes* for animals and cells are listed in Table 1.

DLGAP proteins play an important role in synapse form and function. To assess the relationship between synaptic function and structure during the progression of age‐related AD pathology and *DLGAP2* overexpression, we characterized synaptic properties in patched CA1 pyramidal neurons and then imaged apical and basal dendritic spines. Specifically, we investigated whether overexpression of *DLGAP2* led to alterations in synapse function by measuring synaptic throughput, paired pulse facilitation, and long‐term potentiation (LTP) in CA1 pyramidal cells via stimulation of the Schaffer collateral. We observed no significant effect of *DLGAP2* overexpression on synaptic throughput as measured by input/output curves (Figure 3A). However, on average, brain slices from 14‐month‐old animals required higher stimulation to evoke EPSPs comparable to those from 6‐month‐old animals (F[1, 91] = 23.0, *p* < 0.01) (Figure 3A). Although 5XFAD animals overexpressing *DLGAP2* displayed comparable input/output curves at both 6 and 14 months, an age‐related increase in stimulation required to evoke EPSPs was observed in 5XFAD control injections and Ntg DLGAP2‐overexpressing animals. These results indicate that while *DLGAP2* overexpression does not directly modify synaptic throughput, there may be a synergistic effect of *DLGAP2* expression with AD pathology that minimizes age‐related changes on the strength of synaptic signaling.

To assess alterations in synaptic plasticity, we measured LTP across a 40‐min period. Two‐way repeated‐measures ANOVA detected a significant main effect of AAV construct (F[1, 70] = 4.56, *p* < 0.05), as well as a significant age × AAV construct interaction (F[1, 70] = 7.94, *p* < 0.01). At 6 months, *DLGAP2* overexpression decreased potentiation compared to controls in 5XFAD animals; however, by 14 months, neither controls nor *DLGAP2* overexpressing mice exhibited LTP, regardless of AD status (Figure 3B), possibly because a floor effect prevents measuring diminished LTP past this point. To verify that *DLGAP2* overexpression was not acting presynaptically via expression in CA3 neurons, we measured the PPR. We detected no significant effect of AAV construct, AD status, or age on PPR (Figure S7). These data suggest that overexpression of *DLGAP2* not only fails to strengthen synaptic throughput and LTP but also weakens these outcomes in the presence of AD pathology. There were no significant main effects of *DLGAP2* overexpression, age, or AD status on any of the other intrinsic measurements before or after TBS (Figure S8), suggesting DLGAP2 has specific effects at the synapse and not via general effects on neuron excitability.

### 3.5 DLGAP2 overexpression reduces thin spine density in young animals without AD pathology

To determine whether the observed deficits in synaptic plasticity and subsequent cognitive outcomes from *DLGAP2* overexpression in AD mice could be explained by synaptic structure, we analyzed apical and basal dendritic spine morphologies of patched CA1 pyramidal cells. Spine density was largely comparable between apical and basal dendrites regardless of age, 5XFAD status, or AAV construct. We observed significant main effects of AAV construct (F[1, 293] = 13.84, *p* < 0.001) and age (F[1, 293] = 13.03, *p* < 0.001), as well as an AD status × age interaction (F[1, 293] = 5.71, *p* < 0.05), on dendritic spine density. Although 5XFAD animals showed no significant changes in spine density across any spine type at 6 or 14 months, Ntg animals exhibited a decrease in thin spine density at 6 months of age compared to control injections (Figure 3C,D). However, by 14 months, thin spine density was comparable to both 6‐ and 14‐month eGFP controls, confirming a role for *DLGAP2* in synaptic development and maturation of control mice, although this effect is compensated over the animal’s lifespan. Interestingly, we observed similar outcomes in cultured rat hippocampal cells; by DIV14, cells treated with Dlgap2 cDNA without the presence of amyloid beta had reduced thin spine densities (mean = 1.7, SD = 1.3) compared to GFP‐treated controls (mean = 2.7, SD = 1.1) (Figures 3E, S9). In vitro results highlight the utility of cell culture experiments in studying postsynaptic proteins in a translationally relevant manner. Overall, these results indicate that the effects of DLGAP2 overexpression on behavior and synaptic plasticity in F1 hybrid AD mice are not explained by changes in spine morphology.

## 4 DISCUSSION

This study builds upon our previous work and provides new insights into the role of *DLGAP2* overexpression in the hippocampus within the context of AD pathology. Overexpression of *DLGAP2* had unexpected effects on behavioral outcomes. Whereas we expected overexpression to rescue cognitive deficits in an AD mouse model, we observed exacerbated cognitive decline. Although fully understanding the underlying mechanisms of this effect will require further study, there are several putative mechanisms that may be responsible. Previous work showed that overexpression of certain SHANK isoforms, a PSD protein that interacts with *DLGAP2*, led to manic‐like behavior, including increased locomotor activity and sleep disruption resultant from central nervous system excitatory/inhibitory imbalance.^14^ Although we did not measure sleep patterns in this study, there are reports of sleep disruption in the 5XFAD mouse model,^15^, ^16^ which may explain the marked effects on locomotor activity and cognitive decline in our 5XFAD animals that overexpress *DLGAP2*. However, if *DLGAP2* overexpression does affect sleep quality, the effect on cognition is unclear as this study was unable to determine whether it directly affects cognitive outcomes or if these outcomes result from disruption of sleep.^17^

Similarly, overexpression of Homer1a, an interacting protein with *DLGAP2* and SHANK, in the dorsal hippocampus leads to impaired working memory in mice,^18^ suggesting that regulation of these PSD proteins has significant effects on cognitive outcomes. It has been proposed that overexpression of exogenous proteins may outcompete binding sites for necessary interacting proteins, leading to unexpected mutant phenotypes.^19^ It is very possible that a similar phenomenon is occurring with *DLGAP2* in the PSD, where there are a multitude of tightly regulated proteins interacting with each other. An influx of exogenous *DLGAP2* could very well outcompete other binding sites across the PSD, leading to synaptic destabilization, particularly in both pre‐ and post‐AD‐pathology conditions. DLGAP proteins were previously implicated in synaptic scaling^2^, ^20^ via modifications to interacting PSD proteins. Overexpression‐induced impairment of synaptic scaling offers a promising mechanism through which DLGAP2 may alter AD‐related cognitive outcomes. Indeed, disruption of synaptic scaling has been shown to impair AD‐related memory outcomes.^21^ Future studies will be required to better understand how DLGAP2 interacts with these mechanisms to alter synaptic function and/or network connectivity and how such changes may impact cognitive outcomes.

Given that AD pathology is known to cause dendritic spine loss,^22^, ^23^ it is interesting that we observed spine loss in 6‐month Ntg animals overexpressing *DLGAP2* and not their 5XFAD counterparts. There is clearly an unexpected interaction between AD status and *DLGAP2* overexpression in which presymptomatic AD pathology factors prevent the initial disruption of spine maturation caused by overexpression of *DLGAP2*. Since we see a loss of these spines only in apical and not basal dendrites, a possible explanation is that *DLGAP2* overexpression may hinder function of CA3 neurons, resulting in a reduction of CA1 postsynaptic activity and dendritic spine development in Ntg animals. Presymptomatic soluble Aβ has been shown to induce selective hyperexcitability in the hippocampus, which could overcome the deficits induced by *DLGAP2* overexpression,^24^, ^25^, ^26^, ^27^ explaining why we see this effect only in Ntg animals. However, loss of thin spine density does not coincide with a significant change in spatial working memory or contextual fear‐related memory outcomes and is largely recovered by 14 months of age, limiting the biological significance of this result.

Hippocampal overexpression of *DLGAP2* had minimal effect on synaptic and intrinsic properties of CA1 pyramidal neurons. Although we observed that 6‐month 5XFAD animals overexpressing *DLGAP2* had diminished LTP compared to GFP‐injected controls, this effect was not present by 14 months and did not coincide with observed cognitive deficits. Based on the LTP results, there is an apparent hyperexcitability phenotype in 6‐month 5XFAD animals that is diminished in *DLGAP2* overexpressing animals and is comparable to age‐matched Ntg controls. However, we did not observe a significant difference in synaptic throughput between *DLGAP2* overexpressing and control animals, suggesting that the observed effects in LTP are not explained by synaptic hyperexcitability alone. It is important to note that expression was modest in CA1, with higher intensity observed in cells originating from CA3/dentate/subiculum and in presynaptic projections from recorded regions; thus, the effects of *DLGAP2* overexpression on LTP in recorded CA1 neurons may act through presynaptic projections from CA3.

Although the use of AAV9 to overexpress DLGAP2 enabled targeted expression in excitatory hippocampal neurons, we cannot rule out nonphysiological protein levels and off‐target effects. Immunohistochemistry and Western blotting confirmed that expression was mostly limited to dorsal CA regions, supporting the specificity of our approach. However, viral methods do not replicate natural timings or regulation of gene expression, which may influence synaptic and behavioral outcomes. Future work using genetic models could better clarify the physiological relevance of DLGAP2 in AD.

A limitation of this study is the exclusive use of female mice. This decision was based on our prior study identifying DLGAP2, which included both sexes in both mouse and human mapping studies but did not find a significant effect of sex in either species. Additionally, there is considerable research showing that female F1 5XFAD mice exhibit more robust AD‐related behavioral and synaptic phenotypes compared to males, enabling greater statistical power and experimental reproducibility. Given that AD progression differs between sexes, future studies are needed to determine whether the effects observed here are consistent in male animals. We also acknowledge that the 5XFAD model does not serve as a model for AD‐related tau pathology. Given the role of the hippocampus in tau propagation^28^ and the known interactions between synaptic scaffolding proteins and tau‐related pathways,^29^, ^30^ future studies will need to assess whether DLGAP2 overexpression alters tau pathology or whether the effects of DLGAP2 overexpression are altered compared to Aβ pathology alone.

Our work has shown that virally‐mediated hippocampal overexpression of *DLGAP2* is not an effective means to ameliorate AD‐related pathology. Although the exact mechanism by which overexpression of *DLGAP2* negatively impacts AD‐related cognition will require further investigation, we have shown that *DLGAP2* does play a role in AD‐related cognitive outcomes.

## CONFLICT OF INTEREST STATEMENT

Catherine Kaczorowski has filed a patent related to *DLGAP2*. Andrew Ouellette and Kristen O'Connell have no conflicts of interest to declare. Author disclosures are available in the supporting information.

## CONSENT STATEMENT

No human subjects were part of this research.

## Supporting information



Supporting Information
