# Supplementary material for: Evaluation of hippocampal DLGAP2 overexpression on cognition, synaptic function, and dendritic spine structure in a translationally relevant AD mouse model
Source: Alzheimers Dement. 2025 Sep 30;21(10):e70728. doi: 10.1002/alz.70728 (PMC12481162; doi:10.1002/alz.70728)
Supplement: Supplementary file 1 — Supporting Information [file ALZ-21-e70728-s001.docx]

ICMJE DISCLOSURE FORM

**Date:**	8/7/2025
**Your Name:**	Catherine Kaczorowski
**Manuscript Title:**	Evaluation of hippocampal DLGAP2 overexpression on cognition, synaptic function, and dendritic spine structure in a translationally relevant AD mouse model
**Manuscript Number (if known):**	ADJ-D-25-01256

In the interest of transparency, we ask you to disclose all relationships/activities/interests listed below that are related to the content of your manuscript. "Related" means any relation with for-profit or not-for-profit third parties whose interests may be affected by the content of the manuscript. Disclosure represents a commitment to transparency and does not necessarily indicate a bias. If you are in doubt about whether to list a relationship/activity/interest, it is preferable that you do so.

The author's relationships/activities/interests should be defined broadly. For example, if your manuscript pertains to the epidemiology of hypertension, you should declare all relationships with manufacturers of antihypertensive medication, even if that medication is not mentioned in the manuscript.

In item #1 below, report all support for the work reported in this manuscript without time limit. For all other items, the time frame for disclosure is the past 36 months.

		Name all entities with whom you have this relationship or indicate none (add rows as needed)	Specifications/Comments (e.g., if payments were made to you or to your institution)
		**Time frame: Since the initial planning of the work**	
1	All support for the present manuscript (e.g., funding, provision of study materials, medical writing, article processing charges, etc.) **No time limit for this item.**	☐ None	
		This work was supported by the National Institute of Aging (NIA): F31 AG077860-01A1 (ARO), the University of Maine's Transdisciplinary Predoctoral Training in Biomedical Science and Engineering T32 GM132006 (ARO), NIA RF1 AG063755 (CCK), NIA RF1 AG059778 (KMSO and CCK).	
			Click the tab key to add additional rows.
		**Time frame: past 36 months**	

		Name all entities with whom you have this relationship or indicate none (add rows as needed)	Specifications/Comments (e.g., if payments were made to you or to your institution)
**2**	Grants or contracts from any entity (if not indicated in item #1 above).	☐ **None**	
		This work was supported by the National Institute of Aging (NIA): F31 AG077860-01A1 (ARO), the University of Maine's Transdisciplinary Predoctoral Training in Biomedical Science and Engineering T32 GM132006 (ARO), NIA RF1 AG063755 (CCK), NIA RF1 AG059778 (KMSO and CCK).	
**3**	Royalties or licenses	☒ **None**	
**4**	Consulting fees	☒ **None**	
**5**	Payment or honoraria for lectures, presentations, speakers bureaus, manuscript writing or educational events	☒ **None**	

		Name all entities with whom you have this relationship or indicate none (add rows as needed)	Specifications/Comments (e.g., if payments were made to you or to your institution)
**6**	Payment for expert testimony	☒ **None**	
**7**	Support for attending meetings and/or travel	☐ **None**	
		This work was supported by the National Institute of Aging (NIA): F31 AG077860-01A1 (ARO), the University of Maine's Transdisciplinary Predoctoral Training in Biomedical Science and Engineering T32 GM132006 (ARO), NIA RF1 AG063755 (CCK), NIA RF1 AG059778 (KMSO and CCK).	
**8**	Patents planned, issued or pending	☐ **None**	
		DLGAP2 as a Therapeutic Target for and Alzheimer's Disease and Age-Related Cognitive Decline, Inventors, **Kaczorowski C,** WO2020092862A1, 05/07/2020, PCT/US2019/059311, Inventors: Catherine Kaczorowski, Sarah Neuner; Current Assignee: The Jackson Laboratory	
**9**	Participation on a Data Safety Monitoring Board or Advisory Board	☐ **None**	
		National Institutes on Aging Reserve and Resilience Non-Human Studies Workgroup, NIA, Other, Advisory Member	Resilience/Resistance Against Alzheimer's Disease in Centenarians and Offspring (RADCO), NIA, Other, Scientific Advisor
		Special Mouse Strains Resource P40OD011102, The Jackson Laboratory, External Advisory Board Member	Alzheimer's Disease Sequencing Project (ADSP), NIH, Advisor
		Functional Genomics Consortium, External Advisory Board, NIA, Scientific Advisor	Cognitive Aging Summit IV, Planning Committee, NIH, Member
**10**	Leadership or fiduciary role in	☐ **None**	

		Name all entities with whom you have this relationship or indicate none (add rows as needed)	Specifications/Comments (e.g., if payments were made to you or to your institution)
	other board, society, committee or advocacy group, paid or unpaid	American Federation for Aging Research (AFAR) Board of Directors, Member	
**11**	Stock or stock options	☒ **None**	
**12**	Receipt of equipment, materials, drugs, medical writing, gifts or other services	☒ **None**	
**13**	Other financial or non-financial interests	☒ **None**	

Please place an "X" next to the following statement to indicate your agreement:

☒ I certify that I have answered every question and have not altered the wording of any of the questions on this form.

ICMJE DISCLOSURE FORM

Date: 8/7/2025

Your Name: Andrew Ouellette

Manuscript Title: Evaluation of hippocampal DLGAP2 overexpression on cognition, synaptic function, and dendritic spine structure in a translationally relevant AD mouse model

Manuscript Number (if known): ADJ-D-25-01256

In the interest of transparency, we ask you to disclose all relationships/activities/interests listed below that are related to the content of your manuscript. "Related" means any relation with for-profit or not-for-profit third parties whose interests may be affected by the content of the manuscript. Disclosure represents a commitment to transparency and does not necessarily indicate a bias. If you are in doubt about whether to list a relationship/activity/interest, it is preferable that you do so.

The author's relationships/activities/interests should be defined broadly. For example, if your manuscript pertains to the epidemiology of hypertension, you should declare all relationships with manufacturers of antihypertensive medication, even if that medication is not mentioned in the manuscript.

In item #1 below, report all support for the work reported in this manuscript without time limit. For all other items, the time frame for disclosure is the past 36 months.

		Name all entities with whom you have this relationship or indicate none (add rows as needed)	Specifications/Comments (e.g., if payments were made to you or to your institution)
		**Time frame: Since the initial planning of the work**	
1	All support for the present manuscript (e.g., funding, provision of study materials, medical writing, article processing charges, etc.) No time limit for this item.	☐ None	
		This work was supported by the National Institute of Aging (NIA): F31 AG077860-01A1 (ARO), the University of Maine's Transdisciplinary Predoctoral Training in Biomedical Science and Engineering T32 GM132006 (ARO), NIA RF1 AG063755 (CCK), NIA RF1 AG059778 (KMSO and CCK).	
			Click the tab key to add additional rows.
		**Time frame: past 36 months**	

		Name all entities with whom you have this relationship or indicate none (add rows as needed)	Specifications/Comments (e.g., if payments were made to you or to your institution)
**2**	Grants or contracts from any entity (if not indicated in item #1 above).	☐ **None**	
		This work was supported by the National Institute of Aging (NIA): F31 AG077860-01A1 (ARO), the University of Maine's Transdisciplinary Predoctoral Training in Biomedical Science and Engineering T32 GM132006 (ARO), NIA RF1 AG063755 (CCK), NIA RF1 AG059778 (KMSO and CCK).	
**3**	Royalties or licenses	☒ **None**	
**4**	Consulting fees	☒ **None**	
**5**	Payment or honoraria for lectures, presentations, speakers bureaus, manuscript writing or educational events	☒ **None**	

		Name all entities with whom you have this relationship or indicate none (add rows as needed)	Specifications/Comments (e.g., if payments were made to you or to your institution)
6	Payment for expert testimony	☒ **None**	
7	Support for attending meetings and/or travel	☐ **None**	
		Institute of Aging (NIA): F31 AG077860-01A1 (ARO), the University of Maine's Transdisciplinary Predoctoral Training in Biomedical Science and Engineering T32	
8	Patents planned, issued or pending	☒ **None**	
9	Participation on a Data Safety Monitoring Board or Advisory Board	☒ **None**	
10	Leadership or fiduciary role in other board, society, committee or advocacy group, paid or unpaid	☒ **None**	
11	Stock or stock options	☒ **None**	
12	Receipt of equipment, materials, drugs, medical writing, gifts or other services	☒ **None**	

		Name all entities with whom you have this relationship or indicate none (add rows as needed)	Specifications/Comments (e.g., if payments were made to you or to your institution)
**13**	Other financial or non-financial interests	☒ **None**	

Please place an "X" next to the following statement to indicate your agreement:

☒ I certify that I have answered every question and have not altered the wording of any of the questions on this form.

ICMJE DISCLOSURE FORM

**Date:**	8/7/2025
**Your Name:**	Kristen O'Connell
**Manuscript Title:**	Evaluation of hippocampal DLGAP2 overexpression on cognition, synaptic function, and dendritic spine structure in a translationally relevant AD mouse model
**Manuscript Number (if known):**	ADJ-D-25-01256

In the interest of transparency, we ask you to disclose all relationships/activities/interests listed below that are related to the content of your manuscript. "Related" means any relation with for-profit or not-for-profit third parties whose interests may be affected by the content of the manuscript. Disclosure represents a commitment to transparency and does not necessarily indicate a bias. If you are in doubt about whether to list a relationship/activity/interest, it is preferable that you do so.

The author's relationships/activities/interests should be defined broadly. For example, if your manuscript pertains to the epidemiology of hypertension, you should declare all relationships with manufacturers of antihypertensive medication, even if that medication is not mentioned in the manuscript.

In item #1 below, report all support for the work reported in this manuscript without time limit. For all other items, the time frame for disclosure is the past 36 months.

		**Name all entities with whom you have this relationship or indicate none (add rows as needed)**	**Specifications/Comments (e.g., if payments were made to you or to your institution)**
		Time frame: Since the initial planning of the work	
1	All support for the present manuscript (e.g., funding, provision of study materials, medical writing, article processing charges, etc.)<br>**No time limit for this item.**	☐ None	
		This work was supported by the National Institute of Aging (NIA): F31 AG077860-01A1 (ARO), the University of Maine's Transdisciplinary Predoctoral Training in Biomedical Science and Engineering T32 GM132006 (ARO), NIA RF1 AG063755 (CCK), NIA RF1 AG059778 (KMSO and CCK).	
			Click the tab key to add additional rows.
		Time frame: past 36 months	

		Name all entities with whom you have this relationship or indicate none (add rows as needed)	Specifications/Comments (e.g., if payments were made to you or to your institution)
**2**	Grants or contracts from any entity (if not indicated in item #1 above).	☐ **None**	
		This work was supported by the National Institute of Aging (NIA): F31 AG077860-01A1 (ARO), the University of Maine's Transdisciplinary Predoctoral Training in Biomedical Science and Engineering T32 GM132006 (ARO), NIA RF1 AG063755 (CCK), NIA RF1 AG059778 (KMSO and CCK).	
**3**	Royalties or licenses	☒ **None**	
**4**	Consulting fees	☒ **None**	
**5**	Payment or honoraria for lectures, presentations, speakers bureaus, manuscript writing or educational events	☒ **None**	

		Name all entities with whom you have this relationship or indicate none (add rows as needed)	Specifications/Comments (e.g., if payments were made to you or to your institution)
**6**	Payment for expert testimony	☒ **None**	
**7**	Support for attending meetings and/or travel	☐ **None**	
		This work was supported by the National Institute of Aging (NIA): F31 AG077860-01A1 (ARO), the University of Maine's Transdisciplinary Predoctoral Training in Biomedical Science and Engineering T32 GM132006 (ARO), NIA RF1 AG063755 (CCK), NIA RF1 AG059778 (KMSO and CCK).	
**8**	Patents planned, issued or pending	☒ **None**	
**9**	Participation on a Data Safety Monitoring Board or Advisory Board	☒ **None**	
**10**	Leadership or fiduciary role in other board, society, committee or advocacy group, paid or unpaid	☒ **None**	

		Name all entities with whom you have this relationship or indicate none (add rows as needed)	Specifications/Comments (e.g., if payments were made to you or to your institution)
**11**	Stock or stock options	☒ **None**	
**12**	Receipt of equipment, materials, drugs, medical writing, gifts or other services	☒ **None**	
**13**	Other financial or non-financial interests	☒ **None**	

Please place an "X" next to the following statement to indicate your agreement:

☒ I certify that I have answered every question and have not altered the wording of any of the questions on this form.
